# Tuberous sclerosis: a survey in the canton of Vaud, Switzerland

**DOI:** 10.3389/fmed.2024.1513619

**Published:** 2024-12-12

**Authors:** Olivia Hagon-Nicod, Florence Fellmann, Jan Novy, Sébastien Lebon, Christian Wider, Romain Lazor, Olivier Bonny

**Affiliations:** ^1^Department of Medicine, Service of Internal Medicine, Fribourg State Hospital, Fribourg, Switzerland; ^2^The Collaboratory, University of Lausanne, Lausanne, Switzerland; ^3^Department of Medicine, Service of Neurology, Lausanne University Hospital, Lausanne, Switzerland; ^4^Unit of Pediatric Neurology and Neurorehabilitation, Woman-Mother-Child Department, Lausanne University Hospital, Lausanne, Switzerland; ^5^Department of Medicine, Service of Respiratory Medicine, Lausanne University Hospital, Lausanne, Switzerland; ^6^Department of Medicine, Service of Nephrology, Fribourg State Hospital, Fribourg, Switzerland; ^7^Department of Medicine, Service of Nephrology, Lausanne University Hospital, Lausanne, Switzerland

**Keywords:** tuberous sclerosis complex, mTOR inhibitors, epilepsy, subependymal nodules, subependymal giant cell astrocytomas, angiomyolipomas, lymphangioleiomyomatosis

## Abstract

**Aim of the study:**

Tuberous sclerosis complex (TSC) is a genetic and multisystemic disorder that affects between 1/6’000 and 1/10’000 of newborns. Clinical criteria and/or genetic analysis establish the diagnosis. The mechanistic target of rapamycin (mTOR) inhibitors everolimus or sirolimus reduce the severity of several TSC-related clinical traits. We report here on the epidemiology and management of TSC patients in a large Swiss canton: the canton of Vaud.

**Method:**

We extracted patient files containing the diagnostic code TSC in 2015 at the Lausanne University hospital (tertiary reference center for a population of about 755’000 people) and in specialized neurological institutions of the same region.

**Results:**

We identified 52 patients with a diagnosis of TSC. The majority of the patients with a pathologic result in genetic testing were positive for a pathogenic variant in the *TSC2* gene, including five cases of contiguous gene deletion syndrome of *TSC2* and *PKD1* causing both polycystic kidney disease and TSC. The most frequent clinical manifestations encountered were affecting the skin (87% of patients), the brain (83%), the heart (46%) and the kidneys (46%). Neuropsychiatric disorders were described in 56% of cases. At the time of data collection (2015), there were 2 patients using systemic mTOR inhibitors and 16 patients using topical mTOR inhibitors for dermatological features. Next, we compared this data with those of large published cohorts. While we found fewer cases than expected for the screened population, demographic as well as genetic data were overall similar to the literature. However, we observed that some clinical manifestations (renal, lung and neuropsychiatric disorders) were less frequently described in our cohort.

**Conclusion:**

This work indicates that TSC and some of its clinical manifestations is under-reported. It raises concern that patients with mild manifestations are often not referred to reference centers with dedicated multidisciplinary group. The follow-up by expert board is instrumental in offering systematic screening of all putatively affected organs and to assess the eligibility for targeted treatment.

## 1 Introduction

Tuberous sclerosis complex (TSC) is a rare genetic disorder that may affect almost any organ. The incidence of the disease is estimated between 1/6’000 and 1/10’000 newborns. The pattern of transmission is autosomal dominant, however, about two thirds of cases are linked to *de novo* mutations. The disease is caused by pathogenic variants in *TSC1* or *TSC2* genes that alter the function of proteins, respectively, called hamartin and tuberin. These two proteins form a heterodimer that regulates cell growth and proliferation through the mechanistic target of rapamycin (mTOR) complex. In TSC, the dimer is not functional and the mTOR cascade is constitutively activated, resulting in the formation of multiples benign tumors (also called hamartoma) throughout the body (1–4).

The clinical manifestations of TSC are broad with highly variable degrees of severity but, overall, the morbidity and mortality of this condition are high. The main affected organs are the brain {tubers/migrational defects, epilepsy, subependymal nodules (SENs) or subependymal giant cell astrocytomas (SEGAs)}, the kidneys {angiomyolipomas (AMLs), cysts}, the skin {for example angiofibroma, hypomelanic macules}, the lungs {lymphangioleiomyomatosis (LAM), multifocal micronodular pneumocyte hyperplasia (MMPH)}, the heart {rhabdomyomas} and the eyes {retinal hamartomas}. In addition, the patients often have TSC-associated neuropsychiatric disorders (TAND) such as autism spectrum disorders, intellectual disability, attention deficit/hyperactivity disorders or anxiety disorders (5–9).

For years, only symptomatic treatments were available. However, advances in the understanding of the role of the mTOR pathway in TSC have led to the use of mTOR inhibitors (10, 11). Everolimus is approved for patients with SEGAs when surgery is not possible or when the tumor is invasive, bilateral or multiple (12, 13). Moreover, everolimus is approved for patients with renal AMLs, reducing the tumor size and preventing spontaneous rupture and retroperitoneal bleeding, an event which occurs in large AMLs and may be life-threatening (14–16). In 2018, mTOR inhibitors were approved in Switzerland for patients with intractable epilepsy due to its effect in reducing seizures frequency (17–19). Pulmonary LAM is also stabilized by mTOR inhibitors (20, 21), and an effect of everolimus has also been observed on MMPH (22). For skin lesions, the use of topical mTOR inhibitors is preferred if no systemic indication is warranted (23). However, everolimus is not effective in TAND (24).

The Canton of Vaud in Switzerland has a population of about 755′000 people and its University hospital is a tertiary reference center for the whole state. No data on the number of patients suffering from TSC or on their follow-up and treatment are available in the covered region. We aimed at finding patients followed up in the state and at describing the demography and clinical characteristics. Our second aim was to identify patients that would benefit from mTOR inhibitors treatment.

## 2 Methods

The Ethics Committee of the State approved the protocol. Given the observational nature of the study, the need for informed consent was waived. The data were anonymized. We asked the archiving center to extract the charts of all patients carrying the diagnosis of TSC found in electronic archives of the Lausanne University hospital in 2015. In parallel, the physicians in charge of TSC patients who are part of the TSC board of the Lausanne University hospital (regrouping specialists of the pediatric and adult ages for each of the organs involved in TSC) contributed to patients identification as well as specialized neurologic institutions of the Canton of Vaud where patients with epilepsy and/or intellectual disability might be living (especially the Institution de Lavigny).

We used the modified criteria of Gomez to ascertain that the patients met the diagnosis of TSC (6). We collected information on gender, age, ethnicity, involved organs, genetic analysis and treatments received.

Descriptive statistics were used as appropriate. For age, we used the median value and standard deviation as the distribution of our population was asymmetrical. The prevalence was calculated according to the population of the state in 2015. When no mention was made in the file of a manifestation or a treatment, we inferred that the patient did not suffer from the said manifestation or received the said treatment.

## 3 Results

### 3.1 Demography

**Figure 1:**
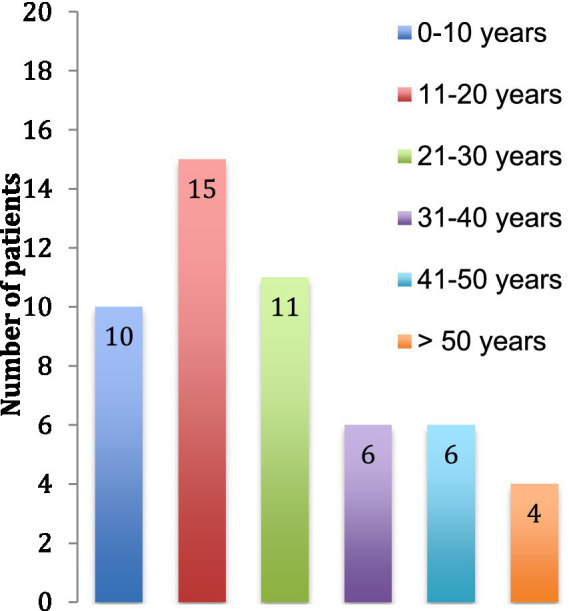
Age of the patients at the time of the study.

We identified 52 patients with a diagnosis of TSC in 2015 in the State of Vaud, Switzerland in which lives a population of about 755′000 persons. Prevalence is thus about 1/15′000. The median age at the time of data collection of the patients was 23 (±16) years (Figure 1) and the majority of patients were females (60%).

### 3.2 Clinical presentation

**Figure 2:**
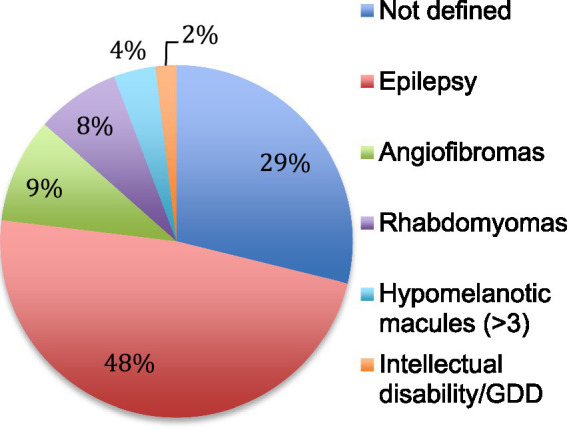
First manifestation of TSC. The first clinical manifestation of TSC is reported in percentage. Not defined means that the initial manifestation was not retrieved in the patient chart. GDD: global developmental delay.

The first manifestations of TSC retrieved in the patient charts were epilepsy, cardiac rhabdomyomas and facial angiofibromas (Figure 2). They appeared at a median age of 9 months (± 6 years). The diagnosis of TSC was established at a median age of 5 years (± 9 years) usually when the addition of other features than the initial manifestation raised the suspicion of a systemic and more complex disorder. In 59% of cases, TSC was recognized as the initial diagnosis but in 41% of the cases, patients were first categorized under various forms of epilepsy.

We further analyzed the pattern of organ involvement. The most frequent manifestations were neurologic (47 patients, 90%), including neuropsychiatric disorders, followed by dermatologic (45 patients, 87%), cardiac (24 patients, 46%) and renal (24 patients, 46%). Lung manifestations, found in 13% (7 patients) of the cohort, developed later than the others, at a mean age of 30 years and were reported as LAM, lung cysts, MMPH and, in one case, pneumothorax.

**Figure 3:**
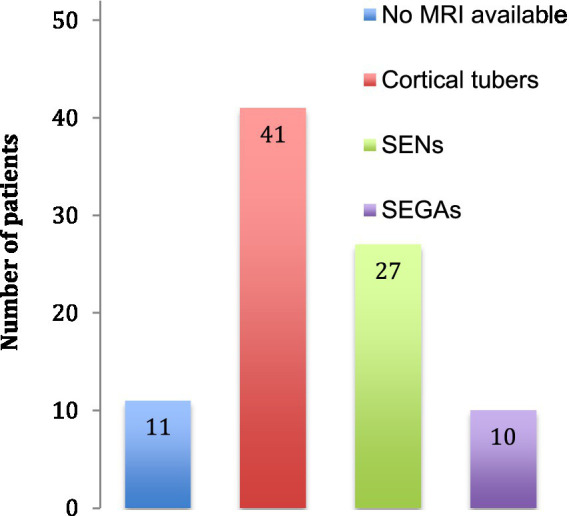
Cerebral abnormalities. The number of patients identified with cerebral abnormalities is indicated. No MRI was available for 11 patients. SENs: subependymal nodules, SEGAs: subependymal giant cell astrocytomas.

The neurological features consisted in structural brain abnormalities, TAND and epilepsy. On 41 brain MRI available, we found 41 with tubers, 27 with SENs and 10 with SEGAs (Figure 3). The TSC-associated neuropsychiatric disorders we found were intellectual disabilities in 28 patients and autism in 5 patients. Regarding epilepsy, the patients often had focal epilepsy or infantile epileptic spasms syndrome.

Regarding renal phenotypic presentation, high blood pressure was reported in 7 patients of the cohort and chronic renal disease in 6 patients. Ophthalmologic, oral, hepatic and splenic manifestations were also described, but less frequently (Table 1).

**Table 1:** Clinical manifestations.

Organ involvement	Number of patients	Median age of appearance in years (SD)
TAND	29/52 (56%)	N/A
Intellectual disability/GDD	28/52 (56%)	N/A
Autism	5/52 (10%)	N/A
Neurologic	43/52 (83%)	
Epilepsy	36/52 (69%)	0.5 (2)
SENs	27/52 (52%)	4.5 (9)
SEGAs	10/52 (19%)	4.5 (9)
Skin	45/52 (87%)	
Hypomelanic macules	36/52 (69%)	1 (6.5)
Angiofibromas	36/52 (69%)	5 (5.5)
Shagreen patch	13/52 (25%)	7 (9)
Cardiac rhabdomyomas	24/52 (46%)	0.5 (3)
Lung	7/52 (13%)	
LAM	5/52 (10%)	30 (N/A)
MMPH	6/52 (11%)	23 (10)
Lung cysts	2/52 (4%)	30 (N/A)
Pneumothorax	1/52 (2%)	N/A
Kidney	24/52 (46%)	
Angiomyolipomas	21/52 (40%)	10 (4.5)
Polycystic kidney	5/52 (10%)	5 (3.5)

SD: Standard deviation; TAND: TSC-associated neuropsychiatric disorders; GDD: global developmental delay; SEN: subependymal nodules; SEGA: subependymal giant cell astrocytomas; LAM: lymphangioleiomyomatosis; MMPH: multifocal micronodular pneumocyte hyperplasia.

### 3.3 Treatments

**Figure 4:**
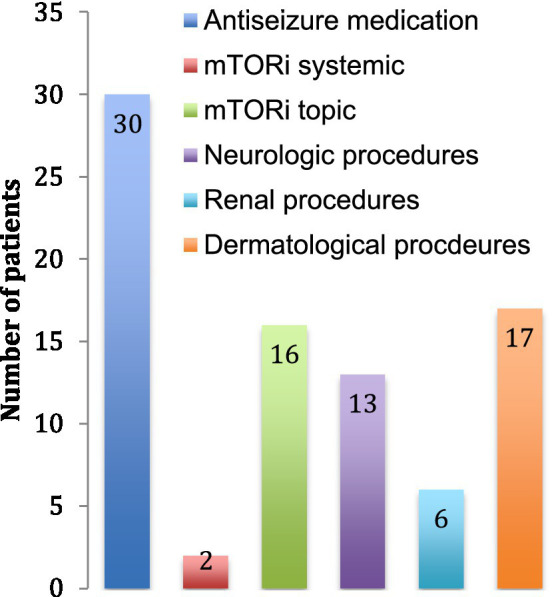
Treatment received. The number of patients receiving each treatment is indicated. Neurological procedures include tumor resections, lobectomies and ventriculo-peritoneal shunts. Renal procedures include arterial embolization, nephrectomy and renal transplantation. Dermatological procedures include laser treatments and excisions of skin tumors.

The main treatment category used was antiseizure medication with one to four drug classes combined. At the time of data collection, there were only 2 patients using systemic mTOR inhibitors, but 16 used topical mTOR inhibitors for dermatological purposes. Thirteen patients (25%) had surgery for neurological problems (symptomatic SEGAs or refractory epilepsy) and 6 patients (11%) had selective renal artery embolization or nephrectomy for AML (Figure 4).

### 3.4 Genetics

**Figure 5:**
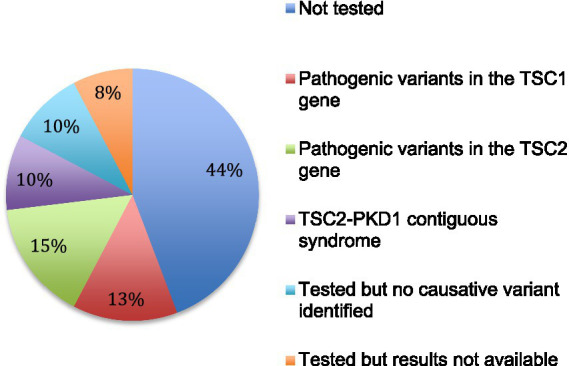
Genetic testing. The results of genetic analysis for the 52 patients are indicated.

Genetic analysis was performed in 29 patients (56%). Results were available for 25 patients. In 5 patients, no pathogenic variant was identified. Fifteen pathogenic variants were identified by sequencing analysis, 8 in the *TSC2* gene and 7 in the *TSC1* gene. In addition, a contiguous gene deletion syndrome (deletion of both *TSC2* and *PKD1* genes, causing both TSC and polycystic kidney disease) was identified in 5 cases (Figure 5).

Five patients had children at the time of data collection. Of these 5 children, 4 were genetically tested and only one was carrying the parental variant.

## 4 Discussion

Due to the unfortunate passing of one of the main investigators, publication of our results has been delayed. Nevertheless, we believe that our findings provide an insight of interest into the epidemiology of tuberous sclerosis patients in the canton of Vaud, Switzerland. However, it should be borne in mind that treatment recommendations have changed between 2015 and now, with a broadening of the indications for mTORi that give an explanation to the low number of patients treated by mTORi in this study.

This work is the first one that attempts to identify all patients suffering from TSC in a dedicated state of Switzerland. We searched for TSC patients in a tertiary university hospital and in specialized institutions hosting patients with epilepsy or patients with severe cognitive impairment, thus probably enriching our cohort with the most severe cases.

According to the published estimated prevalence of the disease (1/6′000–1/10′000), we expected to find 75 to 126 patients with TSC in the State of Vaud (according to the total population in 2015), but found only 52. One explanation for this gap might be that some patients might have been missed by our search. We checked for patients in the electronic files of a tertiary university hospital and in one specialized institution. However, some patients might have been classified under other diagnosis. Alternatively, patients with milder forms of TSC might have been followed-up by physicians in private practice (dermatologists, neurologists, pneumologists, and nephrologists) or in other institutions or hospitals. Altogether, it appears that this cohort probably underestimate the real TSC population and might be enriched with the most severe cases. These findings may also be the results of the absence of a centralized management until this assessment, with a proportion of patients being followed by local specialists.

We compared these results obtained in Switzerland with previously published data obtained from the International Tuberous Sclerosis Complex Consensus Conference of 2012, updated in 2021 (6, 7, 25).

Our demographical data matched the previously published data reporting epilepsy as first manifestation, followed by cardiac rhabdomyomas (26–28). A delay between the first suspicion of TSC and the actual TSC diagnosis is reported in the literature (7,14), and was 2 years on average in our cohort. This illustrates the diagnostic wandering that is typical for rare disorders, with an early putative diagnosis of TSC that is confirmed only later with the appearance of additional manifestations.

The number of familial forms found in our cohort is similar to previously published data (3). However, the parent-to-child transmission observed in our population was lower than expected, probably due to the limited size of our cohort. Moreover, some familial cases were probably missed by our survey, as mildly affected parents or cases of mosaicism might have been undiagnosed.

In our cohort, only 29 patients (56%) had genetic analysis. This may be related to the timing of the study, which was when genetic testing was not generalized and was difficult to get reimbursed by health insurances in Switzerland. We infer that if more tests would be done routinely, more diagnosis would be ascertained and more cases with unclear clinical presentation would be amenable to diagnosis.

Genetic analysis confirmed the diagnosis in 80% of cases (20/25), which is similar to the data in the literature (2, 7). However, in our cohort, the proportion of cases linked to a variant of the *TSC2* gene was higher than expected. The high proportion of contiguous deletions of the *TSC2/PKD1* gene could be linked to the contribution of nephrologists in this work.

**Figure 6:**
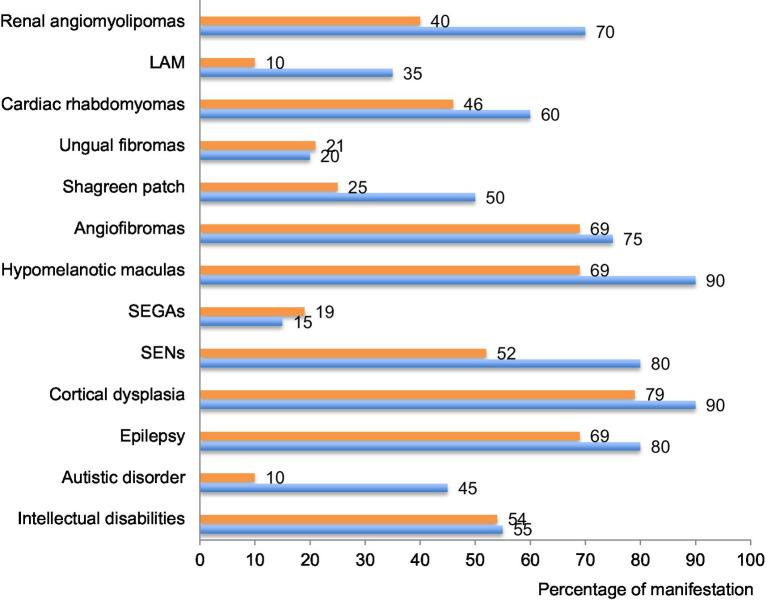
Percentage of each clinical manifestation in our cohort compared to international data. The percentage of patients suffering of each of the described clinical manifestation of the disease is compared between this cohort (in orange) and data provided by the international consortium in 2012 (7) (in blue). TAND: TSC-associated neuropsychiatric disorders; SEN: subependymal nodules; SEGA: subependymal giant cell astrocytomas; LAM: lymphangioleiomyomatosis.

As illustrated on Figure 6, the phenotypes identified in our cohort were overall comparable to previously published data on TSC (6, 7). However, we noted a few remarkable exceptions with lung and renal manifestations as well as shagreen patches and autistic disorders that were less often described in our database compared to others. Regarding lung manifestations, the under-reporting may result from the lack of symptoms at early stages of the disease and from the lack of systematic screening at that time (29, 30). The under-reporting of some clinical manifestations reflects the past management of TSC patients who were not screened for all organ manifestations at that time. These data call for improvement in the management of TSC patients and for systematic screening of all organs annually, according to the updated international TSC recommendations (14). After transition from childhood to adulthood, follow-ups seem less frequent and we noticed that later manifestations of TSC were under-represented in our cohort. This might be due to scarce transition at adolescence. Implementation of transition clinics between childhood and adulthood is expected to bring improvements in the follow-up of adult patients.

Based on this assessment, the Lausanne University hospital has since then launched a multidisciplinary group of specialists including neurologists, neuropediatricians, nephrologists, nephropediatricians, pneumologists, dermatologists and geneticists. This group holds regular meetings to discuss screening, diagnosis and treatment possibilities for TSC patients. This multidisciplinary and trans-age board should provide a better management of patients and help filling the gap noticed in the systematic screening of all organs putatively affected by the disease.

Two patients were started on mTOR inhibitors for SEGA or AML, but when data were censored, the follow-up was too short to draw conclusions on any positive impacts or adverse events of this treatment. More than 30% of our patients were applying topical rapamycin cream on facial angiofibromas with fair efficiency and tolerance.

No death was reported in the medical charts of the patient identified in our cohort. There are only scarce data about the mortality rate of TSC patients in the literature. A Dutch study performed between 1990 and 2012 reported 29 deaths in a total of 351 TSC patients, i.e., a 8% mortality rate over 15 years of surveillance, i.e., five time higher than a randomly selected group of subjects matched for age and gender (31).

This study has limitations. First, this is a small cohort. This may explain why the numbers presented do not always match those found in larger international cohorts. However, the coverage for most severe cases and the description of their phenotype were probably extensive. Second, this study is only retrospective and cross-sectional. Therefore, longitudinal information about the evolution of the patients is incomplete. Finally, the methodology used to identify patients may have introduced a selection bias. Indeed, patients with a mild spectrum of TSC may not have been referred to our center. Conversely, severely affected patients living in institutions for disabled people may not have been all detected.

## 5 Conclusion

This study identified TSC patients in one large state of Switzerland and characterized them. It suggested that the diagnosis of milder forms of TSC probably remains a challenge and that all organs potentially involved should be screened for at the time of diagnosis and a regular basis according to recommendations. It highlighted the need for specific multidisciplinary care as it is offered at the Lausanne University Hospital with the dedicated TSC group. In 2024, with the help of a multidisciplinary management, patients are all evaluated for mTORi treatment and followed up over time.

## Data Availability

The original contributions presented in the study are included in the article/supplementary material, further inquiries can be directed to the corresponding author.

## Author’s note

Part of this work was accomplished during a Master Thesis at the University of Lausanne.

## References

[1] Orphanet: Sclérose tubéreuse de Bourneville. [cited 2016 Dec 6]. Available at: http://www.orpha.net/consor/cgi-bin/OC_Exp.php?Lng=FR&Expert=805

[2] Pfirmann P Combe C Rigothier C. Sclérose tubéreuse de Bourneville: mise au point. Rev Médecine Interne. (2021) 42:714–21. doi: 10.1016/j.revmed.2021.03.003, PMID: 33836894

[3] Northrup H Koenig MK Pearson DA Au KS. Tuberous sclerosis complex. In: Adam MP Feldman J Mirzaa GM Pagon RA Wallace SE Bean LJ., editors. GeneReviews® [Internet]. Seattle (WA): University of Washington, Seattle; (1993) [cited 2023 Dec 3]. Available at: http://www.ncbi.nlm.nih.gov/books/NBK1220/20301399

[4] Zamora EA Aeddula NR. Tuberous sclerosis. In: StatPearls [internet]. Treasure Island (FL): StatPearls Publishing; (2023) [cited 2023 Dec 2]. Available at: http://www.ncbi.nlm.nih.gov/books/NBK538492/

[5] Roach ES Gomez MR Northrup H. Tuberous sclerosis complex consensus conference: revised clinical diagnostic criteria. J Child Neurol. (1998) 13:624–8. doi: 10.1177/088307389801301206, PMID: 9881533

[6] Northrup H Krueger DA. International tuberous sclerosis complex consensus group. Tuberous sclerosis complex diagnostic criteria update: recommendations of the 2012 Iinternational tuberous sclerosis complex consensus conference. Pediatr Neurol. (2013) 49:243–54. doi: 10.1016/j.pediatrneurol.2013.08.001, PMID: 24053982 PMC4080684

[7] Northrup H Aronow ME Bebin EM Bissler J Darling TN de Vries PJ. Updated international tuberous sclerosis complex diagnostic criteria and surveillance and management recommendations. Pediatr Neurol. (2021) 123:50–66. doi: 10.1016/j.pediatrneurol.2021.07.011, PMID: 34399110

[8] Kingswood JC d’Augères GB Belousova E Ferreira JC Carter T Castellana R. TuberOus SClerosis registry to increase disease awareness (TOSCA) – baseline data on 2093 patients. Orphanet J Rare Dis. (2017) 12:2. doi: 10.1186/s13023-016-0553-5, PMID: 28057044 PMC5217262

[9] Portocarrero LKL Quental KN Samorano LP De OZNP Dam R-MMC. Tuberous sclerosis complex: review based on new diagnostic criteria*. An Bras Dermatol. (2018) 93:323–31. doi: 10.1590/abd1806-4841.20186972, PMID: 29924239 PMC6001077

[10] Curatolo P Moavero R. mTOR inhibitors in tuberous sclerosis complex. Curr Neuropharmacol. (2012) 10:404–15. doi: 10.2174/157015912804499537 23730262 PMC3520048

[11] Luo C Ye WR Shi W Yin P Chen C He YB. Perfect match: mTOR inhibitors and tuberous sclerosis complex. Orphanet J Rare Dis. (2022) 17:106. doi: 10.1186/s13023-022-02266-0, PMID: 35246210 PMC8895788

[12] Franz DN Belousova E Sparagana S Bebin EM Frost MD Kuperman R. Long-term use of Everolimus in patients with tuberous sclerosis complex: final results from the EXIST-1 study. PLoS One. (2016) 11:e0158476. doi: 10.1371/journal.pone.0158476, PMID: 27351628 PMC4924870

[13] Franz DN Leonard J Tudor C Chuck G Care M Sethuraman G. Rapamycin causes regression of astrocytomas in tuberous sclerosis complex. Ann Neurol. (2006) 59:490–8. doi: 10.1002/ana.20784, PMID: 16453317

[14] Bissler JJ Kingswood JC Radzikowska E Zonnenberg BA Belousova E Frost MD. Everolimus long-term use in patients with tuberous sclerosis complex: four-year update of the EXIST-2 study. PLoS One. (2017) 12:e0180939. doi: 10.1371/journal.pone.0180939, PMID: 28792952 PMC5549893

[15] Bissler JJ Kingswood JC Radzikowska E Zonnenberg BA Frost M Belousova E. Everolimus for angiomyolipoma associated with tuberous sclerosis complex or sporadic lymphangioleiomyomatosis (EXIST-2): a multicentre, randomised, double-blind, placebo-controlled trial. Lancet. (2013) 381:817–24. doi: 10.1016/S0140-6736(12)61767-X 23312829

[16] Franz DN Budde K Kingswood JC Belousova E Sparagana S de Vries PJ. Effect of everolimus on skin lesions in patients treated for subependymal giant cell astrocytoma and renal angiomyolipoma: final 4-year results from the randomized EXIST-1 and EXIST-2 studies. J Eur Acad Dermatol Venereol JEADV. (2018) 32:1796–803. doi: 10.1111/jdv.14964, PMID: 29569806

[17] French JA Lawson JA Yapici Z Ikeda H Polster T Nabbout R. Adjunctive everolimus therapy for treatment-resistant focal-onset seizures associated with tuberous sclerosis (EXIST-3): a phase 3, randomised, double-blind, placebo-controlled study. Lancet Lond Engl. (2016) 388:2153–63. doi: 10.1016/S0140-6736(16)31419-2, PMID: 27613521

[18] Franz DN Lawson JA Yapici Z Ikeda H Polster T Nabbout R. Adjunctive everolimus therapy for tuberous sclerosis complex-associated refractory seizures: results from the postextension phase of EXIST-3. Epilepsia. (2021) 62:3029–41. doi: 10.1111/epi.17099, PMID: 34693520

[19] Wiegand G May TW Ostertag P Boor R Stephani U Franz DN. Everolimus in tuberous sclerosis patients with intractable epilepsy: a treatment option? Eur J Paediatr Neurol EJPN Off J Eur Paediatr Neurol Soc. (2013) 17:631–8. doi: 10.1016/j.ejpn.2013.06.002 23845174

[20] McCormack FX Inoue Y Moss J Singer LG Strange C Nakata K. Efficacy and safety of sirolimus in lymphangioleiomyomatosis. N Engl J Med. (2011) 364:1595–606. doi: 10.1056/NEJMoa1100391, PMID: 21410393 PMC3118601

[21] Goldberg HJ Harari S Cottin V Rosas IO Peters E Biswal S. Everolimus for the treatment of lymphangioleiomyomatosis: a phase II study. Eur Respir J. (2015) 46:783–94. doi: 10.1183/09031936.00210714, PMID: 26113676

[22] Daccord C Nicolas A Demicheli R Chehade H Hottinger AF Beigelman C. Effect of everolimus on multifocal micronodular pneumocyte hyperplasia in tuberous sclerosis complex. Respir Med Case Rep. (2020) 31:101310. doi: 10.1016/j.rmcr.2020.101310, PMID: 33312857 PMC7720070

[23] Dill PE De Bernardis G Weber P Lösch U. Topical everolimus for facial angiofibromas in the tuberous sclerosis complex. A first case report. Pediatr Neurol. (2014) 51:109–13. doi: 10.1016/j.pediatrneurol.2014.02.016, PMID: 24810875

[24] Krueger DA Sadhwani A Byars AW de Vries PJ Franz DN Whittemore VH. Everolimus for treatment of tuberous sclerosis complex-associated neuropsychiatric disorders. Ann Clin Transl Neurol. (2017) 4:877–87. doi: 10.1002/acn3.494, PMID: 29296616 PMC5740257

[25] Krueger DA Northrup H. International tuberous sclerosis complex consensus group. Tuberous sclerosis complex surveillance and management: recommendations of the 2012 international tuberous sclerosis complex consensus conference. Pediatr Neurol. (2013) 49:255–65. doi: 10.1016/j.pediatrneurol.2013.08.002 24053983 PMC4058297

[26] Staley BA Vail EA Thiele EA. Tuberous sclerosis complex: diagnostic challenges, presenting symptoms, and commonly missed signs. Pediatrics. (2011) 127:e117–25. doi: 10.1542/peds.2010-0192, PMID: 21173003 PMC3010088

[27] Datta AN Hahn CD Sahin M. Clinical presentation and diagnosis of tuberous sclerosis complex in infancy. J Child Neurol. (2008) 23:268–73. doi: 10.1177/0883073807309250 18230839

[28] Ebrahimi-Fakhari D Mann LL Poryo M Graf N von Kries R Heinrich B. Incidence of tuberous sclerosis and age at first diagnosis: new data and emerging trends from a national, prospective surveillance study. Orphanet J Rare Dis. (2018) 13:117. doi: 10.1186/s13023-018-0870-y, PMID: 30016967 PMC6050673

[29] Seibert D Hong CH Takeuchi F Olsen C Hathaway O Moss J. Recognition of tuberous sclerosis in adult women: delayed presentation with life-threatening consequences. Ann Intern Med. (2011) 154:806–294. doi: 10.7326/0003-4819-154-12-201106210-00008, PMID: 21690595 PMC3367307

[30] Miyake M Tateishi U Maeda T Kusumoto M Satake M Arai Y. Pulmonary lymphangioleiomyomatosis in a male patient with tuberous sclerosis complex. Radiat Med. (2005) 23:525–7. PMID: 16485546

[31] Eijkemans MJC van der Wal W Reijnders LJ Roes KCB van Waalwijk van Doorn-Khosrovani SB Pelletier C. Long-term follow-up assessing renal angiomyolipoma treatment patterns, morbidity, and mortality: an observational study in tuberous sclerosis complex patients in the Netherlands. Am J Kidney Dis. (2015) 66:638–45. doi: 10.1053/j.ajkd.2015.05.016, PMID: 26165440

